# Predicting presenteeism using measures of health status

**DOI:** 10.1007/s11136-021-02936-9

**Published:** 2021-07-27

**Authors:** Cheryl Jones, Katherine Payne, Alexander Thompson, Suzanne M. M. Verstappen

**Affiliations:** 1 Manchester Centre for Health Economics, The University of Manchester, 4.306, Jean McFarlane Building, Manchester, M13 9PL UK; 2 Arthritis Research UK-MRC Centre for Musculoskeletal Health and Work, Southampton, UK; 3 Arthritis Research UK Centre for Epidemiology, Division of Musculoskeletal and Dermatological Sciences, School of Biological Sciences, Faculty of Biology, Medicine and Health, The University of Manchester, Manchester Academic Health Science Centre, Manchester, UK; 4 NIHR Manchester Biomedical Research Centre, Central Manchester University Hospitals NHS Foundation Trust, Manchester Academic Health Science Centre, Manchester, UK

**Keywords:** Presenteeism, Autoimmune, Health-related quality of life, Mapping, Prediction, Health status

## Abstract

**Objectives:**

To identify whether it is feasible to develop a mapping algorithm to predict presenteeism using multiattribute measures of health status.

**Methods:**

Data were collected using a bespoke online survey in a purposive sample (*n* = 472) of working individuals with a self-reported diagnosis of Rheumatoid arthritis (RA). Survey respondents were recruited using an online panel company (ResearchNow). This study used data captured using two multiattribute measures of health status (EQ5D-5 level; SF6D) and a measure of presenteeism (WPAI, Work Productivity Activity Index). Statistical correlation between the WPAI and the two measures of health status (EQ5D-5 level; SF6D) was assessed using Spearman’s rank correlation. Five regression models were estimated to quantify the relationship between WPAI and predict presenteeism using health status. The models were specified based in index and domain scores and included covariates (age; gender). Estimated and observed presenteeism were compared using tenfold cross-validation and evaluated using Root mean square error (RMSE).

**Results:**

A strong and negative correlation was found between WPAI and: EQ5D-5 level and WPAI (*r* = − 0.64); SF6D (*r* =− 0.60). Two models, using ordinary least squares regression were identified as the best performing models specifying health status using: SF6D domains with age interacted with gender (RMSE = 1.7858); EQ5D-5 Level domains and age interacted with gender (RMSE = 1.7859).

**Conclusions:**

This study provides indicative evidence that two existing measures of health status (SF6D and EQ5D-5L) have a quantifiable relationship with a measure of presenteeism (WPAI) for an exemplar application of working individuals with RA. A future study should assess the external validity of the proposed mapping algorithms.

**Supplementary Information:**

The online version contains supplementary material available at 10.1007/s11136-021-02936-9.

## Introduction

Paid productivity is conceptualised by two distinct but related concepts: absenteeism and presenteeism. Absenteeism refers to the loss of productivity caused by being *absent* from work because of poor health [1]. Presenteeism describes the impact on productivity whilst *at work* because of health problems [2]. Presenteeism is broadly interpreted as ‘health-related productivity lost whilst at paid work’ [3]. This interpretation suggests a strong conceptual link between health status and presenteeism. Social pressures and other behavioural factors drive people to come into work even when they are not in ‘full health’ [4]. Presenteeism affects not only the individual in poor health but may also have consequences for co-workers where they must pick up the additional workload.

The debate concerning the inclusion of the impact of productivity in economic evaluations continues. Some jurisdictions, such as the National Health Care Institute (Zorginstituut Nederland, ZIN) in the Netherlands, encourage the inclusion of productivity in economic evaluations [5] but others, including, the National institute for health and care excellence (NICE) in England, explicitly exclude it [6]. Normative arguments largely centre on the distribution of consequences as a result of including or excluding productivity in economic evaluations. It is argued that the inclusion of productivity in economic evaluations may influence funding decisions towards healthcare interventions aimed at particular patient subgroups across the population [7, 8].

Driven by methods guidelines, the most commonly used method of economic evaluation has become Cost-effectiveness analysis (CEA) [6, 9]. The method of CEA is often implemented by identifying the consequence of interest as the impact on health status measured using the EQ5D [https://euroqol.org/] and valued using published preference weights [10] to generate Quality-adjusted life years (QALYs) for the relevant population. The lack of ‘gold’ standard methods for identifying, measuring, and valuing the impact of presenteeism may have discouraged researchers from collecting such data further limiting its availability in existing datasets. Prospective studies may be set-up to collect presenteeism related data; however, conducting such studies is almost always an expensive venture. From a pragmatic perspective, an alternative approach may be possible to develop predictions models for presenteeism based on data already collected, for example health status [11].

Since 2005, two studies have quantified the link between health status (measured using the EQ5D) and presenteeism [11, 12]. Lamers et al. [12] used data from a cluster Randomised control trial (RCT), designed to evaluate the effectiveness of physiotherapy guidelines for low back pain in a sample of 483 Dutch patients, to assess the relationship between health status and presenteeism caused by low back pain. Health status was measured using the EQ5D-3L (applying UK preference weights), absenteeism using the Health and labour questionnaire (HLQ) [13], and presenteeism using the Quality and quantity (QQ) method that reports efficiency loss [14]. The analysis estimated a mean EQ5D-3L score of 0.48 for individuals who reported absenteeism (off work for a full 2 weeks) and a mean EQ5D-3L score of 0.71 for those who did not report absence from work. Patients who reported zero days absent from work had a mean efficiency loss due to back pain of 0.20. The authors concluded the study provided evidence that indicated a potential relationship between health status and productivity exists with lower mean EQ5D-3L scores for those reporting absences from work compared to those who did not; however, the evidence was insufficient to recommend the prediction of presenteeism using health status.

In a later study by Krol et al. [11], two distinct prediction model linking health status (EQ5D-3L) with productivity (two separate models for absenteeism and presenteeism) were developed based on responses from a sample of 1013 employed individuals from the Dutch population. Individuals were presented with 16 EQ5D-3L health states and asked to state their *expected* (imagined) level of productivity for each given health state. The subsequent prediction model for presenteeism, measured using the Quality and quantity (QQ) method, was estimated using Generalised estimating equations (GEEs). The purpose of the prediction model was to estimate levels of presenteeism and populate datasets that have not recorded such data. To promote wide applicability of the model across multiple datasets only age and gender were included as covariates in the prediction model [14]. Krol and colleagues [11] assessed the external validation of their prediction model using data collected by Lamers and colleagues [12] and found the model was poor at estimating presenteeism at the individual level but was reasonable when data were aggregated.

Prediction models or ‘mapping’ (also called ‘crosswalking’) algorithms have been produced to develop a quantitative link between non-preference-based, disease-specific measures and generic preference-based measures such as the EQ5D-(3L or 5L) [15]. Franklin and colleagues [16] used mapping methods to quantify the relationship between health status, measured using the EQ5D-3L, and capability, measured using the ICEpop CAPability measure for Older people (ICECAP-O). The study concluded that a clear relationship could not be defined [16]. Nevertheless, the methods used by Franklin and colleagues did indicate the potential for the development of a mapping algorithm that: (1) uses health status as an explanatory variable; and (2) maps from health to a concept beyond health.

An important recommendation for analysts seeking to develop a mapping algorithm is that the first step should be to understand whether there is sufficient conceptual overlap between the constructs being mapped [17]. There is existing qualitative evidence to support that there is conceptual validity between existing measures of health status (the EQ5D and SF6D) and the concept of presenteeism. Jones and colleagues used the results from qualitative semi-structured interviews to show a conceptual link between the impact on health status, as measured by the EQ5D or SF6D, and the potential impact on presenteeism [18]. The study did not, however, provide a quantifiable link between the two concepts of health status and presenteeism providing motivation for the development of a mapping algorithm [18]. The aim of this study was to identify whether it is feasible to develop a mapping algorithm that can be used to predict presenteeism using existing multiattribute measures of health status. The goals of the mapping algorithms are twofold: (1) is to explore the extent to which health status/capability measures are able to predict presenteeism, allowing for a further understanding between any potential relationship; and (2) to provide a method which allows presenteeism to be retrospectively predicted using health status/capability data in large datasets where such data have not been collected.

## Methods

### Case study

Rheumatoid arthritis (RA) is a fluctuating chronic inflammatory auto-immune condition that primarily causes stiffness and pain in joints and tendons of the hands and feet. It is the most common inflammatory auto-immune condition in the United Kingdom (UK) and if left untreated can cause permanent damage to joints leaving the individual disabled [19]. Typically, disease onset occurs before the age of 65 years old (the current retirement age in the UK) meaning that individuals are frequently affected during their working lifetime [20]. There is substantial evidence to suggest that RA is significantly associated with increases in presenteeism [21].

### Study sample

The relevant study population for this study was defined as adults who were currently in work and had a self-reported diagnosis of RA. A sample of adults (18 years and over) with RA who were currently working in full-time or part-time paid positions were invited to take part in the study. The study sample was identified and recruited using an internet panel provider (ResearchNow, now called Dynata; https://www.dynata.com/). A sample size of *n* = 500 was informed in line with published mapping studies listed on the Health economics research centre (HERC) database of mapping studies (version 7.0) [22].

### Data collection

Data were collected using a bespoke online survey. Ethical approval from the University of Manchester was granted (reference number: 16144). Informed consent was taken at the beginning of the survey before the participant completed the survey. Respondents were informed they could leave the survey at any time without providing a reason; however, it was also explained that once the participant clicked “submit” they would not be able to retrieve and withdraw their responses due to anonymisation. The survey collected data on each individual’s: demographics; job type (sedentary, light, medium and heavy) and employment status (full-time, part-time; employed or self-employed); disease severity, measured by the Routine assessment of patient index data three survey (RAPID3 [23]; medications; health status (EQ5D-5L and SF6D); and presenteeism, measured using the Work productivity activity impairment (WPAI) [24]. The WPAI was selected as the relevant measure of presenteeism for this study because it is recommended for use in patients with RA by the Outcomes measures in rheumatology group (OMERACT) [25], adopts a patient perspective, and is relatively short, thereby reducing participant burden. The WPAI asks: ‘During the past 7 days, how much did your rheumatoid arthritis affect your productivity whilst you were working?’. The WPAI records levels of presenteeism using a zero to ten Likert scale where zero indicates ‘RA had no effect on my work’ and ten indicates ‘RA completely prevented me from working’. The WPAI has been well tested for its validity and reliability both within RA and other chronic conditions [26, 27]. The EQ5D-5L and SF6D were transformed into index values using the relevant published algorithms available and acceptable for use during the analysis period of this study [28, 29].

### Analysis

Data analysis involved three stages in line with published recommendations for producing mapping algorithms [30, 31].

### Statistical correlation

Spearman’s rank (*r*) correlation was used to measure the strength and direction between the measures of health status (EQ5D-5L/SF6D) and presenteeism (WPAI). The potential strength of the correlation was described by categories defined prior to the start of the study: very weak (*r* = 0 to 0.19); weak (*r* = 0.2 to 0.39); moderate (*r* = 0.40 to 0.59); strong (*r* = 0.6 to 0.79); and very strong (*r* = 0.8 to 1) [32]. If a sufficient correlation (defined as moderate or above) was identified between the EQ5D-5L [33] and/or SF6D [34] with WPAI [24] then those measures of health status would be taken forward and developed to form a mapping algorithm for presenteeism. Supplementary Appendix 1 describes the approach to understand the performance of the WPAI in this study sample in terms of reliability (internal consistency) measured using Cronbach’s alpha.

### Regression model and specification

Potentially suitable regression methods for producing a mapping algorithm were defined prior to analysis of the data. The dependent variable was defined as the level of presenteeism (using WPAI) and the independent variables included a measure of health status (EQ5D-5L or SF6D) with covariates for age and gender. This study took a parsimonious approach to the inclusion of additional covariates to allow for wider applicability of a specified mapping algorithm; a method used in published algorithms [11, 35]. Age was collected in pre-defined age bands and gender (male; female) was treated as a dummy variable.

There are many potential regression models that can be used to generate a mapping algorithm. Published guidelines for developing a mapping algorithm state that the selection of model type depends on the characteristics of the dependent variable (categorical, ordinal, etc.) and its distribution [30]. Longworth and Rowen [30] explain the need to take into account the bi-model distribution of the EQ5D for algorithms attempting to predict EQ5D values. However, the focus of this study is to develop an algorithm that predicts levels of presenteeism and not utilities for the EQ5D. Presenteeism, measured using the WPAI, can take values from zero to ten, increasing by increments of one and typically exhibits a negative distribution skewed to the left (many zeros). No formal guidelines exist to inform the model type for predicting presenteeism, therefore five types of regression models were selected as potential candidates to develop the mapping algorithm: (1) Ordinary Least Squares (OLS); (2) Tobit; (3) Censored Least Absolute Deviation (CLAD); (4) Ordinal Logit (Ologit); (5) multi-variable logit (mlogit).

OLS models a linear relationship and assumes equal distance between values of the dependent variable; this is consistent with the interpretation of the levels (zero to ten) included in the WPAI. OLS is an unbounded regression model and may produce inconsistent estimators when dealing with censored (left or right) dependent variables [36]. Tobit models are a potentially useful alternative when data are censored. Tobit models allow the analyst to set upper and lower limits for the dependent variable, for example 0 ≤ *y* ≤ 10. Tobit models are highly sensitive to heteroscedasticity which can lead to inconsistent estimates and affecting the standard errors [37]. Therefore, the use of a CLAD model was explored because it is less sensitive to skewed data and is robust to heteroscedasticity but is also censored at a lower value of zero [38].

Ordinal logit regression models are used for its ability to predict an ordinal dependent variable, for which presenteeism, as measured by the WPAI, is in this study. Ordinal logit models estimate the cumulative probability of observing an outcome using specified explanatory variables. The multinomial logit model, a similar regression model to ordinal logit where it also uses cumulative probabilities to predict an outcome level, was selected for its ability to generate predictions across multiple outcome levels. The observed outcome of the WPAI may take one of multiple levels ranging from zero to ten.

Six model specifications (see Table 1) were run for each of the five regression models. In total, 60 potential mapping models were specified to test their ability to predict presenteeism. The EQ5D-5L and SF6D were incorporated into separate mapping models as: (1) index scores; and (2) dummy variables for each level of severity associated with each domain.

**Table 1:** Model specifications

Health status	Health status information	Covariates
EQ5D-5L	Index Score	–
SF6D	Index Score	–
EQ5D-5L	Index Score	Age, Gender
SF6D	Index Score	Age, Gender
EQ5D-5L	Index Score	Age*Gender
SF6D	Index Score	Age*Gender
EQ5D-5L	Domain level using dummies	–
SF6D	Domain level using dummies	–
EQ5D-5L	Domain level using dummies	Age, Gender
SF6D	Domain level using dummies	Age, Gender
EQ5D-5L	Domain level using dummies	Age*Gender
SF6D	Domain level using dummies	Age*Gender

### Model performance

The Root mean square error (RMSE) was used as the metric from which to judge models relative ability to predict presenteeism; a lower RMSE reflects smaller prediction errors. The RMSE was selected as the measures of prediction accuracy because it is able to penalise to a greater extent those predictions that are further away from the actual observed value [39]. The RMSE is an appropriate measure of error where predicted levels of presenteeism that are further away from the actual are interpreted to be considerably worse compare to those that are closer to the true value. The Mean bias error (MBE) is used to estimate the average bias, under or over-prediction, of the model as defined by the sign (negative or positive) and may be used to inform measures to correct to the bias [40].

To calculate the RMSE and MBE for each model, the K-fold method was used to split the sample. There is no ‘gold standard’ method for selecting the most appropriate number of folds, however ten folds is common practice [41] and therefore *K* = 10 in this study. The RMSE results are reported using graphical plots and across quartiles of the WPAI’s range.

## Results

A total of 514 individuals completed the survey. A total of 42 individuals were dropped from the sample. The primary outcome, level of presenteeism as measured by the WPAI, was missing for 42 observations (8% of the total sample).

Dynata (ResearchNow) recommend rejecting surveys where participants take less than 33% of the average time taken to complete the survey; participants completed the survey within an average of equating to 4.29 min. Therefore, a further 13 observations were dropped from the sample because they completed the entire questionnaire in less than 4.29 min. Two observations were dropped because they reported contradicting answers to two separate questions that asked them about their current work status. One observation reported to be on maternity leave; and one reported to have worked longer hours than are available in one week. The final sample consisted of 472 individuals working with RA. Table 2 describes the key characteristics of the study sample.

**Table 2:** Key characteristics of sample

Characteristics	*n*	(%)
Total	472	(100)
Gender, female	297	(63)
Age bands, years		
18 – 34	47	(10)
35 – 39	50	(11)
40 – 44	47	(10)
45 – 49	68	(14)
50 – 54	89	(19)
55 – 59	81	(17)
60+	90	(19)
Full-time employee	325	(69)
Non-manual	255	(54)
Manual	70	(15)
Part-time employee	132	(28)
Non-manual	97	(21)
Manual	35	(7)
Self-employed	15	(32)
Non-manual	14	(3)
Manual	1	(0.002)
Disease severity (RAPID)		
High	236	(50)
Medium	146	(31)
Low/Remission	90	(19)
Medication	183	(39)
Biologics only	32	(7)
csDMARDs only	114	(24)
Biologics and csDMARDS	3	(8)
Health status	Mean	(min, max)
EQ5D	0.683	(− 0.281, 1)
SF6D	0.693	(0. 301, 1)
Presenteeism	Mean	(min, max)
WPAI	3.34	(0, 10)
Missing data	42	(8)

**Fig. 1:**
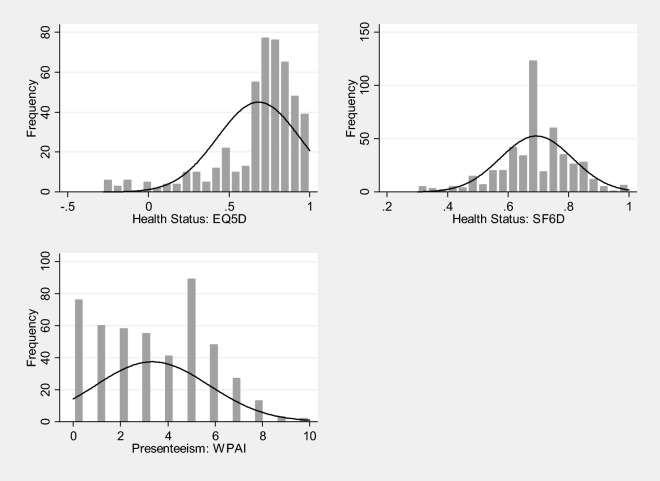
Distribution of health status and presenteeism

Figure 1 illustrates the distributions of two measures of health status (EQ5D-5L or SF6D) and presenteeism (WPAI). The distribution for the EQ5D is highly skewed to the right whereas the distribution of the SF6D appears on visual inspection to be normally distributed. The distribution for presenteeism is slightly skewed to the left and negative; however, there is a spike in the number of people reporting the value of ‘five’ as their level of presenteeism. Testing for heteroscedasticity is reported in the Supplementary Appendix 2. In this study, the internal consistency of WPAI, measured using Cronbach’s alpha, was 0.899 suggesting sufficiently high reliability for this measure in this sample (see Supplementary Appendix 1).

### Statistical correlation

Spearman’s rank correlation suggested a strong and negative correlation between the WPAI and EQ5D-5L (*r* = − 0.64) and the WPAI and SF6D (*r* = − 0.60) providing evidence that, in theory, mapping algorithms could be produced using either of these measures of health status.

### Model selection

Table 3 presents information on the predictive ability (RMSE) of all the models ran to predict presenteeism using EQ5D-5L or SF6D data. The MBE for all models was zero indicating zero bias in the models. Overall, the models that used dummy variables for each of the domains of the EQ5D-5L and SF6D produced more accurate estimates compared with those that used the index score and typically, those models that used covariates (age and gender) also performed better compared with models that did not include covariates.

**Table 3:** RMSE of all potential model specifications for predicting presenteeism

Model number	Health status	Health status (Index or Dummy)	Covariates	Model	Mean ± SD	RMSE	Range RMSE (min; max)
36	SF6D	Dummy	Age*gender	OLS	3.37 ± 0.06	1.7858	1.764 – 1.787
33	EQ5D	Dummy	Age*gender	OLS	3.37 ± 0.07	1.7859	1.760 – 1.811
32	EQ5D	Dummy	Age and gender	OLS	3.34 ± 0.08	1.7979	1.778 – 1.822
35	SF6D	Dummy	Age and gender	OLS	3.34 ± 0.07	1.8039	1.769 – 1.827
31	EQ5D	Dummy	–	OLS	3.36 ± 0.11	1.8060	1.789 – 1.828
34	SF6D	Dummy	–	OLS	3.34 ± 0.06	1.8110	1.776 – 1.838
38	EQ5D	Dummy	Age and gender	Tobit	3.17 ± 0.10	1.8551	1.527 – 2.003
39	EQ5D	Dummy	Age*gender	Tobit	3.15 ± 0.10	1.8593	1.692 – 2.100
37	EQ5D	Dummy	–	Tobit	3.12 ± 0.12	1.8593	1.570 – 2.087
41	SF6D	Dummy	Age and gender	Tobit	3.13 ± 0.09	1.8675	1.730 – 2.101
40	SF6D	Dummy	–	Tobit	3.12 ± 0.09	1.8676	1.707 – 2.094
42	SF6D	Dummy	Age*gender	Tobit	3.16 ± 0.08	1.8729	1.735 – 2.057
6	SF6D	Index	Age*gender	OLS	3.38 ± 0.06	1.9296	1.914 – 1.948
5	SF6D	Index	Age and gender	OLS	3.33 ± 0.08	1.9384	1.923 – 1.958
11	SF6D	Index	Age and gender	Tobit	3.13 ± 0.12	1.9502	1.836 – 2.082
4	SF6D	Index	–	OLS	3.34 ± 0.06	1.9517	1.933 – 1.963
7	EQ5D	Index	–	Tobit	3.16 ± 0.12	1.9562	1.854 – 2.089
10	SF6D	Index	–	Tobit	3.08 ± 0.14	1.9575	1.831 – 2.019
8	EQ5D	Index	Age and gender	Tobit	3.15 ± 0.12	1.9590	1.834 – 2.127
3	EQ5D	Index	Age*gender	OLS	3.36 ± 0.07	1.9609	1.951 – 1.973
12	SF6D	Index	Age*gender	Tobit	3.12 ± 0.12	1.9649	1.894 – 2.097
2	EQ5D	Index	Age and gender	OLS	3.38 ± 0.06	1.9702	1.953 – 1.980
44	EQ5D	Dummy	Age and gender	CLAD	3.22 ± 0.07	1.9717	1.655 – 2.186
1	EQ5D	Index	–	OLS	3.35 ± 0.07	1.9732	1.957 – 1.987
43	EQ5D	Dummy	–	CLAD	3.14 ± 0.09	1.9794	1.660 – 2.494
9	EQ5D	Index	Age*gender	Tobit	3.18 ± 0.12	1.9798	1.829 – 2.141
47	SF6D	Dummy	Age and gender	CLAD	3.22 ± 0.11	1.9898	1.763 – 2.258
46	SF6D	Dummy	–	CLAD	3.20 ± 0.05	1.9931	1.761 – 2.247
17	SF6D	Index	Age and gender	CLAD	3.20 ± 0.09	2.0680	1.795 – 2.375
16	SF6D	Index	–	CLAD	3.15 ± 0.15	2.0727	1.876 – 2.465
13	EQ5D	Index	–	CLAD	3.12 ± 0.13	2.0767	1.934 – 2.246
45	EQ5D	Dummy	Age*gender	CLAD	3.33 ± 0.07	2.0846	1.789 – 2.474
48	SF6D	Dummy	Age*gender	CLAD	3.35 ± 0.06	2.0867	1.977 – 2.223
15	EQ5D	Index	Age*gender	CLAD	3.23 ± 0.11	2.1058	1.828 – 2.662
18	SF6D	Index	Age*gender	CLAD	3.19 ± 0.11	2.1070	1.841 – 2.393
50	EQ5D	Dummy	Age and gender	Ologit	3.21 ± 0.10	2.1700	1.963 – 2.443
49	EQ5D	Dummy	–	Ologit	3.23 ± 0.10	2.1703	1.624 – 2.943
51	EQ5D	Dummy	Age*gender	Ologit	3.13 ± 0.09	2.1704	1.977 – 2.462
53	SF6D	Dummy	Age and gender	Ologit	3.14 ± 0.06	2.1809	1.965 – 2.656
52	SF6D	Dummy	–	Ologit	3.13 ± 0.06	2.1843	1.798 – 2.709
54	SF6D	Dummy	Age*gender	Ologit	3.21 ± 0.06	2.2107	1.926 – 2.631
14	EQ5D	Index	Age and gender	CLAD	3.23 ± 0.11	2.2584	1.836 – 3.253
19	EQ5D	Index	–	Ologit	3.07 ± 0.11	2.3372	2.039 – 2.586
28	SF6D	Index	–	Mlogit	3.37 ± 0.11	2.3468	2.290 – 2.698
20	EQ5D	Index	Age and gender	Ologit	3.00 ± 0.16	2.3598	1.917 – 2.644
24	SF6D	Index	Age*gender	Ologit	3.29 ± 0.05	2.3600	2.039 – 2.595
23	SF6D	Index	Age and gender	Ologit	3.31 ± 0.04	2.3691	2.039 – 2.595
22	SF6D	Index	–	Ologit	3.31 ± 0.04	2.3782	2.039 – 2.316
25	EQ5D	Index	–	Mlogit	3.18 ± 0.06	2.3816	2.083 – 2.783
26	EQ5D	Index	Age and gender	Mlogit	2.97 ± 0.17	2.4067	1.826 – 2.706
29	SF6D	Index	Age and gender	Mlogit	3.37 ± 0.13	2.4257	2.054 – 2.718
21	EQ5D	Index	Age*gender	Ologit	3.02 ± 0.13	2.4454	2.034 – 2.631
55	EQ5D	Dummy	–	Mlogit	3.19 ± 0.09	2.7372	2.364 – 3.727
27	EQ5D	Index	Age*gender	Mlogit	2.97 ± 0.15	2.7544	2.431 – 3.030
30	SF6D	Index	Age*gender	Mlogit	3.15 ± 0.15	2.8326	2.302 – 3.170
56	EQ5D	Dummy	Age and gender	Mlogit	3.19 ± 0.09	2.9051	2.433 – 3.667
58	SF6D	Dummy	–	Mlogit	3.28 ± 0.07	3.4632	2.616 – 4.052
59	SF6D	Dummy	Age and gender	Mlogit	3.33 ± 0.09	3.8356	3.401 – 4.418
57	EQ5D	Dummy	Age*gender	Mlogit	3.30 ± 0.11	3.8413	3.152 – 4..470
60	SF6D	Dummy	Age*gender	Mlogit	3.31 ± 0.14	6.5645	5.293 – 7.775

**Fig. 2:**
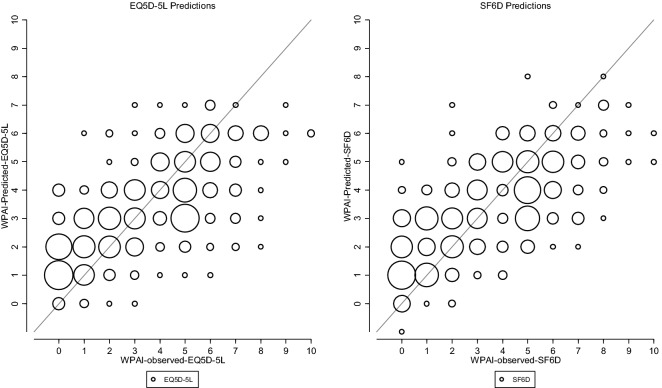
Observed and predicted levels of presenteeism using EQ5D-5L and SF6D. The size of the circles represent the volume of observed and predicted values of presenteeism

Table 3 reports the RMSE for each model. The model with the smallest RMSE (1.7858) was for the OLS model with SF6D dummy model with age and gender interacted (model 36). The model with the next smallest RMSE was the OLS model with EQ5D dummy model with age and gender interacted (model 33) which had a RMSE that was fractionally larger than model 36 (RMSE = 1.7859). The full algorithms for models 36 and 33 are presented in the supplementary appendix 4. The observed and predicted values of the two model specifications (33 and 36) are illustrated in Fig. 2. The graphical plots suggest the two mapping algorithms were able to predict presenteeism scores with some degree of accuracy. However, both models tended to over-predict levels of presenteeism at observed levels between zero and four and under-predict levels of presenteeism at observed levels of five and over.

The RMSE of models 33 and 36 were compared across the quartile of the range of the presenteeism scale (Table 4). Model 36 had lower RMSEs in three of the four quartiles suggesting that, overall, model 36 generates more accurate predictions of presenteeism compared with model 33.

**Table 4:** RMSE across subsets of WPAI (presenteeism) range

WPAI score	Observations per quartile	Model 33 RMSE (EQ5D)	Model 36 RMSE (SF6D)
0 – 1	136	0.4944	0.4826
2 – 3	113	0.4970	0.4830
4 – 5	130	0.4679	0.4681
6 – 10	93	0.9497	0.9366

## Discussion

This study aimed to develop a mapping algorithm that predicts levels of presenteeism, measured by the WPAI, using HRQoL data. The study tested a wide range of potential models. The top six models, based on the lowest RMSE, were similar where they all used OLS regression and dummy variable data. However, given the mean scores and widely overlapping RMSE confidence intervals, based on this current study, it is clear no model outperforms any other. Descriptively, the SF6D domain level dummies, with age and gender interacted (model 36) (the model with the lowest RMSE) would be a potential candidate model that could be tested further as based on the results of this study. With that said, the range of RMSE (minimum and maximum) values for each model do not increase uniformly across all models as the RMSE increases (see Table 3) suggesting the potential need to conduct a study with a larger sample size.

The top two models that utilise the SF6D or EQ5D-5L domain level dummies, with age and gender interacted (model 36 and 33, see Table 3) had only fractionally worse predictive ability, as measured by the RMSE. Examining the graphical plots of the predicted levels of presenteeism estimated by these two models (Fig. 2) reveals little difference in predictions between the two models. It is reasonable to suggest that the SF6D and the EQ5D-5L may have the potential to predict presenteeism to a similar degree of accuracy; a pragmatic result for populating those datasets that house only EQ5D-5L or SF6D data.

The qualitative study that explored the conceptual validity between measures of health status, captured by the SF6D and EQ5D, and presenteeism suggested both measures of health status were able to capture important factor of RA that increase levels of presenteeism [18]. The results of this study suggest the same; however, further research is needed to confirm the predictive ability of the SF6D and EQ5D for levels of presenteeism.

### Strengths

To our knowledge, this is the second of two studies that have applied mapping algorithms to quantitatively link health status with a concept beyond health and is the first to apply mapping methods to predict levels of presenteeism. Prediction models using health status data for presenteeism are limited and have focussed their efforts on developing models using EQ5D-3L data [11, 12], whereas the study presented here is the first to develop a prediction model for presenteeism using EQ5D-5L and SF6D data.

There is strength in the results of this study because they are based on data collected from individuals who were still working with RA during the time of this study. The results capture the reality of working with RA including nuances such as the ability to adapt and manage a chronic condition. This is in direct contrast with the study design used by Krol and colleagues [11] where individuals were asked to imagine their levels of presenteeism given a specific health state. A potential reason why Krol and colleagues [11] did not find an strong relationship between health status and presenteeism is that individual who have no experience of working with a chronic condition have little understanding of the actual impact it may have on their ability to work. However, it must be noted that the results from this study are far from conclusive and an external validation study is needed to confirm confidence in the algorithms generated in this study.

### Limitations

The developed mapping algorithm must be understood in light of some limitations. Few observations for presenteeism at levels 9 and 10 (very severe levels of presenteeism) meant that the mapping algorithm struggled to predict these high levels of presenteeism (see Supplementary Appendix 3).

This preliminary study used a complete case analysis of a dataset comprising data from all completed surveys. We did not use multiple imputation methods to generate estimates of ‘missing’ data because the literature is currently unclear regarding how to combine multiple imputation within predictive modelling. Research into multiple imputation methods is currently very active with researchers exploring issues related to; the assumptions made when applying imputation methods [42], how to account for imputation uncertainty and its impact on subsequent statistical testing [43], and how model selection is affected after having applied multiple imputation methods [44]. Using a complete case analysis approach will not affect the observed estimated mapping algorithms but may affect the generalisability of the results.

Developing a prediction model based on few observations is not recommended, therefore, we considered the possibility of collapsing observed presenteeism levels eight, nine and ten to make one group. Ultimately, this approach was decided against where the primary purpose of the mapping algorithm was to enable a prediction of presenteeism at all levels. It may be the case that the lack of observations for very high levels of presenteeism (nine and ten) reflects the current health and work status of the individuals sampled in this study where those individuals who are able to continue working do so because they know they are, broadly, able to keep pace with their work and therefore report low, mild or moderate levels of presenteeism. Individuals who might report severe presenteeism may be struggling to remain productive at work and are potentially less likely to engage with studies such as this potentially making them a difficult subgroup to reach. Further research is required to study the characteristics of individuals who work with severe levels of presenteeism. Furthermore, the evidence presented in this study may potentially help towards an improved understanding of the differences between inter-individual levels of presenteeism; however, further research is needed to quantify absolute productivity losses. Potential new methods, such as the Productivity adjusted life years (PALYs), as discussed by Ademi et al. [45] aim to quantify productivity loss and incorporate productivity explicitly in cost-effectiveness studies. A mapping algorithm linked to PALY utilities may be useful, particularly to populate those datasets where PALY utilities have not been collected.

To promote the use of a mapping algorithm, it must be rigorously tested using an external dataset [30]. Unfortunately, and to our knowledge, there is no dataset that has SF6D, EQ5D-5L and WPAI data that can be used to externally validate the algorithms.

The mapping algorithms were developed using an RA population only. Further research is needed to understand whether the models could be used in: (1) populations with diseases similar to RA, for example ankylosing spondylitis; and (2) populations with any other form of chronic physical condition that makes working difficult, for example chronic pain.

## Conclusion

The results of this study suggest there is a quantifiable relationship between health status, measured using the EQ5D-5L and SF6D, and presenteeism, measured using WPAI. This study indicates the potential to develop a mapping algorithm to populate large datasets that have health status data, EQ5D-5L or SF6D, but do not currently possess presenteeism data; a pragmatic and inexpensive solution towards generating estimates of presenteeism where such data are scarce. However, it is not possible to recommend the mapping algorithms developed in this study due to the lack of external validity. Further research is needed to assess the external validity and understand the generalisability of the mapping algorithms in populations working with different chronic conditions.

## Supplementary Information

Below is the link to the electronic supplementary material.

Supplementary file1 (DOCX 262 kb)
