# Supplementary material for: Predicting presenteeism using measures of health status
Source: Qual Life Res. 2021 Jul 27;31(2):425–35. doi: 10.1007/s11136-021-02936-9 (PMC8847206; doi:10.1007/s11136-021-02936-9)
Supplement: Supplementary file 1 — Supplementary file1 (DOCX 262 kb) [file 11136_2021_2936_MOESM1_ESM.docx]

Supplementary Appendices for:

Predicting presenteeism using measures of health status. *Quality of Life Research*

Authors: Cheryl Jones, Ph.D,^1,2*^, Katherine Payne, Ph.D^1 ,^ Alexander Thompson^1^, Suzanne M.M. Verstappen, Ph.D^3,4^

Appendix 1: Reliability of the Work Productivity Activity Index for Rheumatoid Arthritis

Appendix 2: Testing for heteroscedasticity

Appendix 3: Number of observations per level of presenteeism

Appendix 4: Suggested mapping algorithms for predicting presenteeism using measures of health status

**Appendix 1: Reliability of the Work Productivity Activity Index for Rheumatoid Arthritis**

*Aim:* to quantify the reliability (internal consistency) of Work Productivity Activity Index for Rheumatoid Arthritis in the study sample.

*Method:* Reliability (or internal consistency) describes the extent to which all the items in a test measure the same concept (or construct). Cronbach’s alpha is a way to quantify the inter-relatedness of the items within the test^[[1]](#footnote-1)^. In this study, the inter-relatedness of two sub-scales in the Work Productivity Activity Index for Rheumatoid Arthritis (WPAI-RA) was assessed: scale 1 and scale 2.

Scale 1

Respondents answer using a ten-point scale where zero represents ‘RA had no effect on my work’ and ten represents ‘RA completely prevented me from working’

Scale 2

Respondents answer using a ten-point scale where zero represents ‘RA had no effect on my activities’ and ten represents ‘RA completely prevented me from my activities’

*Result:* A total of 472 respondents completed both scales in the WPAI-RA. The calculated Cronbach’s alpha between these two scales was 0.899 that suggests a sufficiently high degree of internal consistency based on standard rules of thumb (Cronbach’s alpha between 0.6 and 0.8)^1^.

References for Appendix 1

Tavakol M, Dennick R. Making sense of Cronbach's alpha. *Int J Med Educ*. 2011;2:53-55

**Appendix 2: Testing for Heteroscedasticity**

The presence of heteroscedasticity was tested for using two approaches (i) informally using graphical plots of the residual errors and (ii) formally using the Breusch-Pagan test (Brusch-Pagan et al, 1979). Presenteeism, measured using the work productivity activity impairment (WPAI) questionnaire, and health status, measured using the EQ5D-5L index score, were regressed using ordinary least squares (OLS). The residuals errors were estimated and are presented in Figure A.1 for each measure of health status. Visual inspection of Figure A2.1 suggests there may be systematic bias in the errors.

Figure A2.1: Plots of the Residual errors

Graph on left: Presenteeism (WPAI) and EQ5D-5L index, graph on right Presenteeism (WPAI) and SF6D index

The results from the formal Breusch-Pagan test produce p-values: p =0.4829 (WPAI and EQ5D-5L) and p = 0.7917 (WPAI and SF6D) above the 5% significance levels. The interpretation of these test results mean that the null hypothesis cannot be rejected and the conclusion is that there is constant variance of the errors (no heteroscedasticity). The lack of concurrence between the informal (visual inspection) and formal Breusch-Pagan test drove the decision to estimate models that are sensitive (Tobit) and insensitive (censored least absolute deviations, CLAD) to heteroscedasticity.

References for Appendix 2

Breusch, T.S. and A.R. Pagan, 1979, A simple test for heteroscedasticity and random coefficient variation, Econometrica 47, 1287- 1294.

**Appendix 3: Number of observations for each level of presenteeism**

Table A3.1 shows the number of observations for each observed level of presenteeism as identified and measured by the work productivity activity impairment (WPAI) questionnaire. Presenteeism levels 9 and 10, representing very severe levels of presenteeism (barely able to work), have very few observations.

Table A3.1: Number observations per level of presenteeism (WPAI)

| WPAI | Number of observations | % |
| --- | --- | --- |
| 0 | 76 | 16.10 |
| 1 | 60 | 12.71 |
| 2 | 58 | 12.29 |
| 3 | 55 | 11.65 |
| 4 | 41 | 8.69 |
| 5 | 89 | 18.86 |
| 6 | 48 | 10.17 |
| 7 | 27 | 5.72 |
| 8 | 13 | 2.75 |
| 9 | 3 | 0.64 |
| 10 | 2 | 0.42 |
| Total | 472 | 100 |

**Appendix 4:** Suggested mapping algorithms for predicting presenteeism using measures of health status

This study produced two suggested mapping algorithms to predict presenteeism each using a specific measure of health status: SF6D (see Table A4.1) and EQ5D (see Table A4.2). Table A4.1 presents the mapping algorithm for presenteeism using the Short Form Six Dimensions (SF6D) and included dummy variables with interactions with age and gender. Table A4.2 presents the mapping algorithm for presenteeism using EuroQol Five Dimension- Five Level (EQ5D-5L) survey and included dummy variables with interactions with age and gender.

Table A4.1: Mapping algorithm using SF6D

| **Definition** | **Variable**  **label** | **Coef.** | **95% Conf. Interval** | | **Std. Err.** | **P>t** |
| --- | --- | --- | --- | --- | --- | --- |
| Intercept | Intercept | -0.068 | -1.511 | 1.375 | 0.734 | 0.926 |
| Physical level 2 | sfphys2 | 0.009 | -0.635 | 0.652 | 0.327 | 0.979 |
| Physical level 3 | sfphys3 | -0.295 | -0.975 | 0.386 | 0.346 | 0.396 |
| Physical level 4 | sfphys4 | -0.060 | -0.755 | 0.634 | 0.353 | 0.865 |
| Physical level 5 | sfphys5 | 0.005 | -0.963 | 0.973 | 0.493 | 0.992 |
| Role function level 2 | sfrole2 | 1.118 | 0.634 | 1.602 | 0.246 | 0.000 |
| Role function level 3 | sfrole3 | 1.990 | 1.246 | 2.734 | 0.379 | 0.000 |
| Role function level 4 | sfrole4 | 1.123 | 0.327 | 1.918 | 0.405 | 0.006 |
| Social function level 2 | sfsocial2 | 0.323 | -0.191 | 0.838 | 0.262 | 0.217 |
| Social function level 3 | sfsocial3 | 0.603 | -0.065 | 1.271 | 0.340 | 0.077 |
| Social function level 4 | sfsocial4 | 0.884 | 0.031 | 1.736 | 0.434 | 0.042 |
| Social function level 5 | sfsocial5 | 2.082 | 0.648 | 3.516 | 0.730 | 0.005 |
| Pain level 2 | sfpain2 | 0.724 | -0.212 | 1.660 | 0.476 | 0.129 |
| Pain level 3 | sfpain3 | 1.372 | 0.388 | 2.356 | 0.501 | 0.006 |
| Pain level 4 | sfpain4 | 2.504 | 1.479 | 3.529 | 0.521 | 0.000 |
| Pain level 5 | sfpain5 | 2.816 | 1.677 | 3.955 | 0.579 | 0.000 |
| Pain level 6 | sfpain6 | 3.104 | 1.598 | 4.611 | 0.767 | 0.000 |
| Mental health level 2 | sfmental2 | 0.280 | -0.198 | 0.759 | 0.244 | 0.250 |
| Mental health level 3 | sfmental3 | 0.559 | -0.045 | 1.163 | 0.307 | 0.070 |
| Mental health level 4 | sfmental4 | -0.044 | -0.868 | 0.780 | 0.419 | 0.916 |
| Mental health level 5 | sfmental5 | 1.130 | -0.157 | 2.417 | 0.655 | 0.085 |
| Vitality level 2 | sfvita2 | -0.376 | -1.295 | 0.542 | 0.467 | 0.421 |
| Vitality level 3 | sfvita3 | -0.372 | -1.313 | 0.569 | 0.479 | 0.437 |
| Vitality level 4 | sfvita4 | -0.363 | -1.346 | 0.620 | 0.500 | 0.468 |
| Vitality level 5 | sfvita5 | -0.224 | -1.313 | 0.865 | 0.554 | 0.686 |

Table A4.2: Suggested mapping algorithm using EQ5D-5 level

| **Definition** | **Variable label** | **Coef.** | **95% Conf. Interval** | | **Std. Err.** | **P>t** |
| --- | --- | --- | --- | --- | --- | --- |
| Intercept | Intercept | -0.343 | -1.630 | 0.944 | 0.655 | 0.600 |
| Mobility level 2 | mo2 | 0.858 | 0.388 | 1.328 | 0.239 | > 0.000 |
| Mobility level 3 | mo3 | 0.883 | 0.283 | 1.483 | 0.305 | 0.004 |
| Mobility level 4 and 5 | mo4_5 | 0.597 | -0.291 | 1.485 | 0.452 | 0.188 |
| Self-care level 2 | sc2 | 0.311 | -0.115 | 0.736 | 0.217 | 0.152 |
| Self-care level 3 | sc3 | 0.985 | 0.317 | 1.653 | 0.340 | 0.004 |
| Self-care level 4 and 5 | sc4_5 | 1.068 | -0.141 | 2.277 | 0.615 | 0.083 |
| Usual activities level 2 | ua2 | 0.630 | 0.145 | 1.116 | 0.247 | 0.011 |
| Usual activities level 3 | ua3 | 1.119 | 0.408 | 1.831 | 0.362 | 0.002 |
| Usual activities level 4 and 5 | us4_5 | 2.050 | 0.869 | 3.231 | 0.600 | 0.001 |
| Pain and discomfort level 2 | pd2 | 0.443 | -0.396 | 1.283 | 0.427 | 0.300 |
| Pain and discomfort level 3 | pd3 | 1.104 | 0.209 | 1.100 | 0.455 | 0.016 |
| Pain and discomfort level 4 and 5 | pd4_5 | 1.406 | 0.317 | 2.496 | 0.554 | 0.012 |
| Anxiety and depression level 2 | ad2 | 0.670 | 0.281 | 1.119 | 0.213 | 0.001 |
| Anxiety and depression level 3 | ad3 | 1.100 | 0.580 | 1.610 | 0.262 | > 0.000 |
| Anxiety and depression level 4 and 5 | ad4_5 | 0.783 | 0.094 | 1.472 | 0.351 | 0.026 |

1. [↑](#footnote-ref-1)
